# Perceived diagnostic and system-related barriers to timely referral and interfacility transfer of trauma patients in Namibia: a cross-sectional survey

**DOI:** 10.1016/j.afjem.2026.101026

**Published:** 2026-10-31

**Authors:** Johan Du Plessis, Paulus Ambunda, Andrit Lourens

**Affiliations:** a Department of Clinical Health Sciences, School of Health Sciences, Faculty of Health, Natural Resources and Applied Sciences, Namibia University of Science and Technology, Windhoek, Namibia; b Motor Vehicle Accident Fund of Namibia, Windhoek, Namibia; c Division of Emergency Medicine, Department of Family, Community and Emergency Care, Faculty of Health Sciences, University of Cape Town, Cape Town, Western Cape, South Africa

**Keywords:** Interfacility transfer, "Patient Transfer"[Mesh], Trauma, "Wounds and Injuries"[Mesh], Critically injured

## Abstract

**Introduction:**

Patient transfers aim to ensure continuity of care, but referral-transfer pathway processes are often time-consuming and encounter various delays. This study determined healthcare workers’ perceptions of clinical and non-clinical factors contributing to delays in the transfer process of critically injured trauma patients from the Otjozondjupa District Hospitals to the Windhoek Referral Hospitals and explored mitigation strategies.

**Methods:**

A cross-sectional online survey was conducted in 2023 at three district hospitals, private and parastatal ambulance services in the Otjozondjupa region, Namibia. Healthcare workers employed in Emergency Centres and Prehospital Emergency Care Providers, working in the setting for 6 months or longer, were eligible to participate. We used convenience and snowball sampling.

**Results:**

Of the 50 respondents, 64.0% (n = 32) worked in Emergency Centres. Thirty-nine (n = 39, 78.0%) perceived referral/transfer delays. Diagnostic imaging and medical procedures were perceived clinical factors contributing to delays. Non-clinical/system-related factors included bed, ambulance, and medical doctor availability, a lack of human resources and management protocols, and limited knowledge of existing policies and procedures. Suggested mitigating strategies centred on targeted training needs, improved resource allocation, and enhanced Information Technology systems.

**Conclusion:**

Healthcare workers perceived diagnostic processes, stabilisation procedures, and system-related constraints as barriers to timely interfacility transfer of critically injured trauma patients. Improving time to definitive care may benefit critically injured trauma patients while easing pressure on facilities caring for other urgent patients. These findings may inform local reviews of trauma-transfer processes and support future prospective studies that measure actual transfer times and objectively confirmed causes of delay.

**African Relevance**

• To our knowledge, this is the first Namibian study describing healthcare workers’ perception of delays in referral and interfacility transfer of critically injured trauma patients.
• This study addresses an under-explored area of trauma care in Namibia by describing healthcare workers’ perceptions of factors associated with delays in referral and interfacility transfer of critically injured trauma patients from district hospitals to referral hospitals.
• The findings may be relevant to geographically dispersed, resource-constrained African settings, where long transfer distances, limited specialist services, and reduced local trauma care capacity affect access to definitive care.
• Respondents identified practical barriers, relevant to resource-limited emergency care systems, including ambulance availability, staffing shortages, after-hours diagnostic delays, and hospital bed constraints.
• Understanding these perceived barriers may support locally appropriate interventions, including targeted training, clearer referral and dispatch pathways, improved ambulance readiness and stronger communication between referring and receiving facilities.

## Introduction

Trauma remains a major global public health concern, accounting for an estimated 4.4 million deaths annually [1]. It is the leading cause of mortality among the working population, while 27% of traumatic injuries result in permanent disability [2]. Across Africa, trauma represents a growing concern, particularly secondary to road traffic accidents (RTAs), with the highest RTA-related fatality rate globally (19.6 deaths per 100,000) [3].

Namibia faces a disproportionate burden of road traffic-related deaths. The Cause of Death Report 2016 – 2017 [4] reported that 75% (n = 1053) of trauma-related deaths were due to road traffic injury (RTIs), underscoring the urgent need to strengthen trauma care systems and reduce preventable trauma-related deaths in Namibia.

Critically injured patients require rapid multidisciplinary evaluation and timely definitive care [5]. District hospitals in Namibia, such as those in the Otjozondjupa region, are staffed by general practitioners and provide initial assessment, resuscitation, and stabilisation, but lack the expertise and resources for definitive trauma care. Consequently, seriously injured patients require referral and interfacility transfers (IFTs) to referral hospitals in Windhoek that provide specialised and definitive trauma care. Referral is the decision to refer a patient to a higher level of care [6], whereas an IFT transfers a patient from one medical facility to another and is integral to the healthcare system [7]. According to the Namibian Ministry of Health and Social Services’ (MoHSS) referral policy (2015) [8], IFTs are required when referring facilities cannot provide definitive care. In this study, the term “referral-transfer pathway” will encompass both the referral decision and the IFT process.

Several African and other low- and middle-income country (LMIC) studies have reported delays in referral, transfer and access to definitive injury care, citing barriers such as referral execution, transport availability, staffing, infrastructure constraints and long travel distances to definitive care [[9], [10], [11]]. Regional anecdotal reports suggest that for patients who clearly require IFTs, 3–5 h may lapse from arrival at district hospitals to the initiation of IFT. Including transport time, patients may wait 6–9 h to reach definitive care, potentially compromising outcomes. Delays in the referral-transfer pathway have been categorised as clinical factors (e.g., directly related to patient management) and non-clinical (e.g., demographic, temporal, weather-related and workload) [12].

The study aimed to identify the perceived clinical and non-clinical factors contributing to delays in the referral-transfer pathway for critically injured trauma patients from Otjozondjupa District Hospitals to Windhoek Referral Hospitals in Namibia, to identify mitigating strategies, and to explore whether delays were facility-specific or system-wide.

## Methods

We conducted a descriptive cross-sectional survey to identify perceived clinical and non-clinical factors contributing to delays in the referral-transfer pathway of critically injured patients from Otjozondjupa District Hospitals to referral facilities in Windhoek, Namibia. Open-ended questions explored potential mitigating strategies.

The study was conducted in the Otjozondjupa Region, central Namibia, encompassing 105,185 km² and a population of 220,811 [13]. The region is served by 4 district hospitals: Okahandja, Otjiwarongo (ODH), Okakarara (OKDH), and Grootfontein District Hospital (GDH). However, Okahandja District Hospital was excluded from the study setting due to its proximity to the referral hospitals (69 km). The remaining referring hospitals are between 252 and 462 km from the referral facilities (Fig. 1). The referral hospital is the facility equipped with the necessary expertise and resources to provide definitive treatment for critically injured trauma patients and is also called the receiving hospital.

**Fig. 1:**
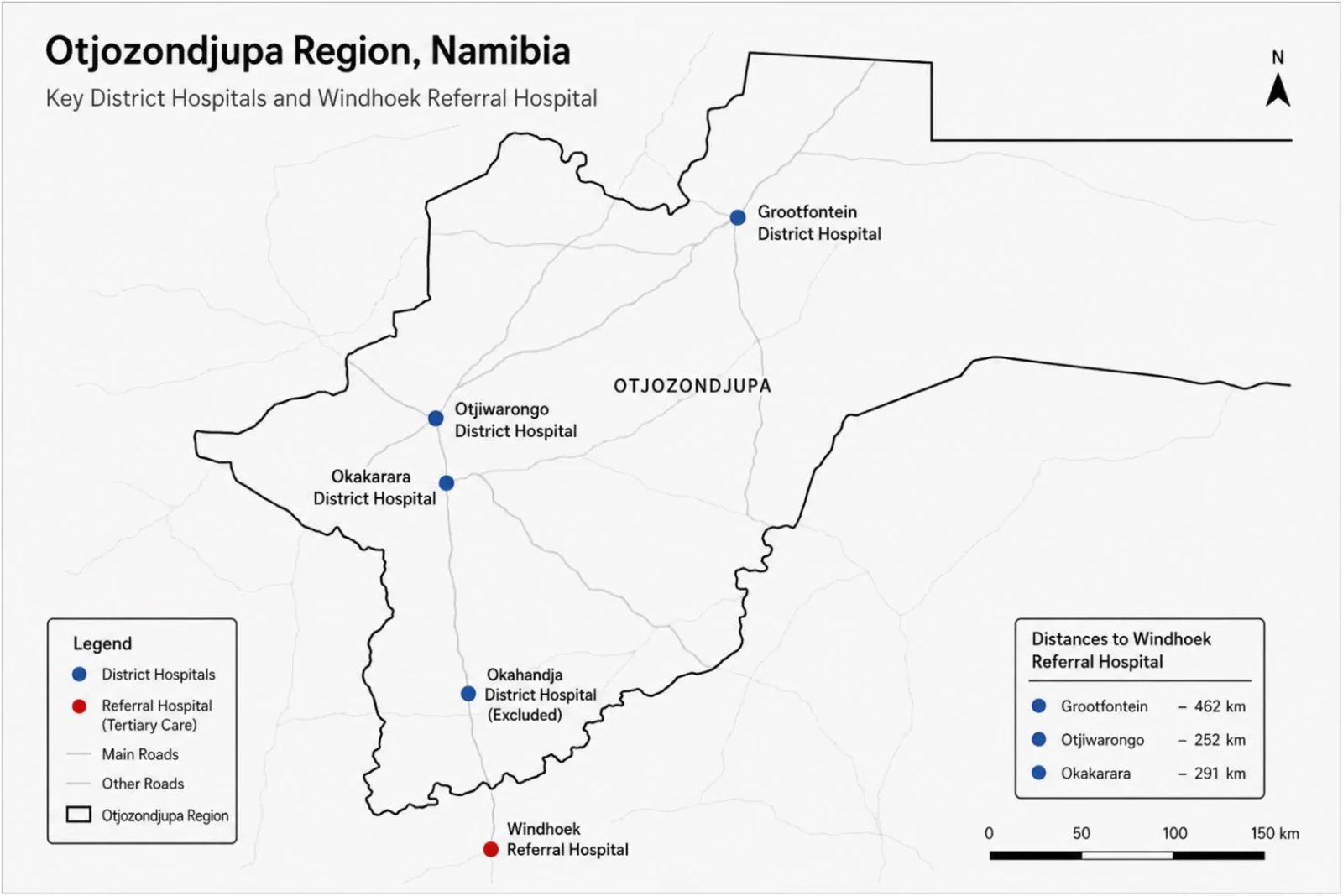
Locations of Otjozondjupa region district hospitals and Windhoek referral hospitals. (Source: Map created by the authors using QGIS, Natural Earth boundary data, and OpenStreetMap road data ©OpenStreetMap contributors. The layout and visual presentation were enhanced using OpenAI ChatGPT (GPT-5.5)).

The Guidelines for the Implementation of the National Referral Policy [8] specify that trauma cases requiring referral include polytrauma, spinal injuries, head injury (moderate and severe), and fractures requiring open reduction and internal fixation. Patient referral begins when the primary healthcare facility determines it cannot provide definitive care. The attending Medical Doctor (MD) initiates the process by contacting the referral facility to confirm that a receiving MD is available. To prepare for transport, the patient undergoes diagnostic testing and initial stabilisation, which, anecdotally, is time-consuming.

IFTs via road are facilitated by state, private and parastatal (a semi-private and state organisation) ambulance services at each town and district hospital. State ambulance services generally operate with a nurse and driver due to the limited availability of prehospital emergency care providers (PECPs) and may include an Advanced Life Support (ALS) Paramedic or an MD for critical cases. Private and parastatal services conduct IFTs based on the patient's level of care and the availability of a fully staffed, appropriately qualified PECP team. Anecdotally, ambulances in the study setting provide both primary response and IFTs.

The study population included Emergency Centre (EC) healthcare workers (MDs, Registered Nurses (RNs) and Enrolled Nurses (ENs)) employed at the three district hospitals, as well as PECPs qualified at the Intermediate Life Support (ILS) level or higher, working in the region for at least six months. Individuals not meeting these criteria were excluded.

Based on an estimated population of 70, a 95% confidence level with a 5% margin of error, and an estimated 50% response rate, the *a priori* calculated sample size was 60 participants [14]. The final sample size of 50 participants, drawn from a population of 70, at a 95% CI, yielded a margin of error of 7.5%.

Non-probability convenience and snowball sampling were used to invite eligible participants over 4 weeks, from 15 September to 13 October 2023.

Data were collected using an online self-administered structured questionnaire (Supplemental Material 1) to assess perceived referral and transfer delays. The questionnaire was based on a study by Nolan et al. [15] and anecdotal evidence from the region. Social desirability bias and respondent fatigue were minimised through balanced wording and concise layout. Factors not related to patient management, such as documentation, logistics, ambulance preparedness, and staff complement, were deemed non-clinical and system-related, whereas those related to patient management were deemed clinical.

Several measures were implemented to enhance internal validity. Construct validity was established by ensuring that the questions were grounded in relevant, existing knowledge and addressed the research objectives. Measuring all aspects of the construct in the questionnaire, including clinical and non-clinical factors and mitigation measures, ensured content validity. Face validity was established through input from five experts and a pilot study. The questionnaire was refined based on feedback..

The questionnaire was distributed online via Microsoft Forms (Microsoft Corporation, Redmond, WA, USA) and in hard copy to participants with no/limited internet access; however, none of the latter were completed.

Data were analysed using IBM SPSS Statistics for Windows, Version 28 (IBM Corp., Armonk, NY, USA), with a statistician's assistance. Descriptive statistics were used to summarise data, including measures of frequency (relative and absolute) for categorical data and measures of central tendency and variability for continuous data.

Relationships between categorical variables were explored using cross-tabulations and the Chi-square test for independence. Statistical significance was set at p < 0.05. Results were presented in text, tables, and graphs.

Ethical approval was obtained from the Namibia University of Science and Technology (NUST) Faculty Research Ethics Committee (FHNRAS:42/2023) and the Research Committee at the MoHSS (Ref: 22/3/1/2). Subsequently, permission to conduct research was sought from relevant authorities in the region. Participation was voluntary and anonymous, with no personal identifiable information collected. Participants provided tick-box informed consent after reading the information sheet (Supplemental Material 1). Participants were informed of their right to withdraw until the online questionnaire was submitted, at which point the data were anonymised.

## Results

Of the 70 potential participants, 50 (71.4% response rate) submitted the completed questionnaire.

Eighteen (36.0%) respondents selected prehospital as work setting, including two nurses (possible incorrect selection), while 32 (64.0%) selected the EC setting (Table 1). Professional representation was broadly distributed across the cadres involved in IFTs, including MDs (n = 13, 26.0%), ENs (n = 12, 24.0%) and PECPs (n = 16, 32.0%).

**Table 1:** Respondents characteristics.

Respondent Characteristics	n (%)
**Gender:**	
Male	19 (38.0%)
Female	31 (62.0%)
**Work Setting:**	
Prehospital	18 (36.0%)
Emergency Centre (EC)	32 (64.0%)
**Work Area:**	
Grootfontein District Hospital (GDH)	10 (20.0%)
Okakarara District Hospital (OKHD)	9 (18.0%)
Otjiwarongo District Hospital (ODH)	20 (40.0%)
Parastatal Ambulance Services	2 (4.0%)
Private Ambulance Services	9 (18.0%)
**Profession:**	
Medical Doctor (MD)	13 (26.0%)
Registered Nurse (RN)	9 (18.0%)
Enrolled Nurse (EN)	12 (24.0%)
Advanced Life Support (ALS) Paramedic	2 (4.0%)
Emergency Care Technician (ECT)	1 (2.0%)
Intermediate Life Support (ILS)	13 (26.0%)
**Total**	**50 (100%)**

Most respondents were female (n = 31, 62%) and working in the current position/department for >24 months (n = 28, 56%). Respondents were drawn from district hospitals and ambulance services across the region, with the largest proportion from ODH (n = 20, 40.0%).

### Frequency of referral-transfer pathway delays

Thirty-nine respondents (78.0%) reported experiencing referral-transfer delays (sometimes, often, and always) (Fig. 2). No statistically significant difference was found between referral-transfer delays (never, rarely, sometimes, often, and always) of critically injured patients and the medical facility (χ2 = 10.276, df = 16, p = 0.852).

**Fig. 2:**
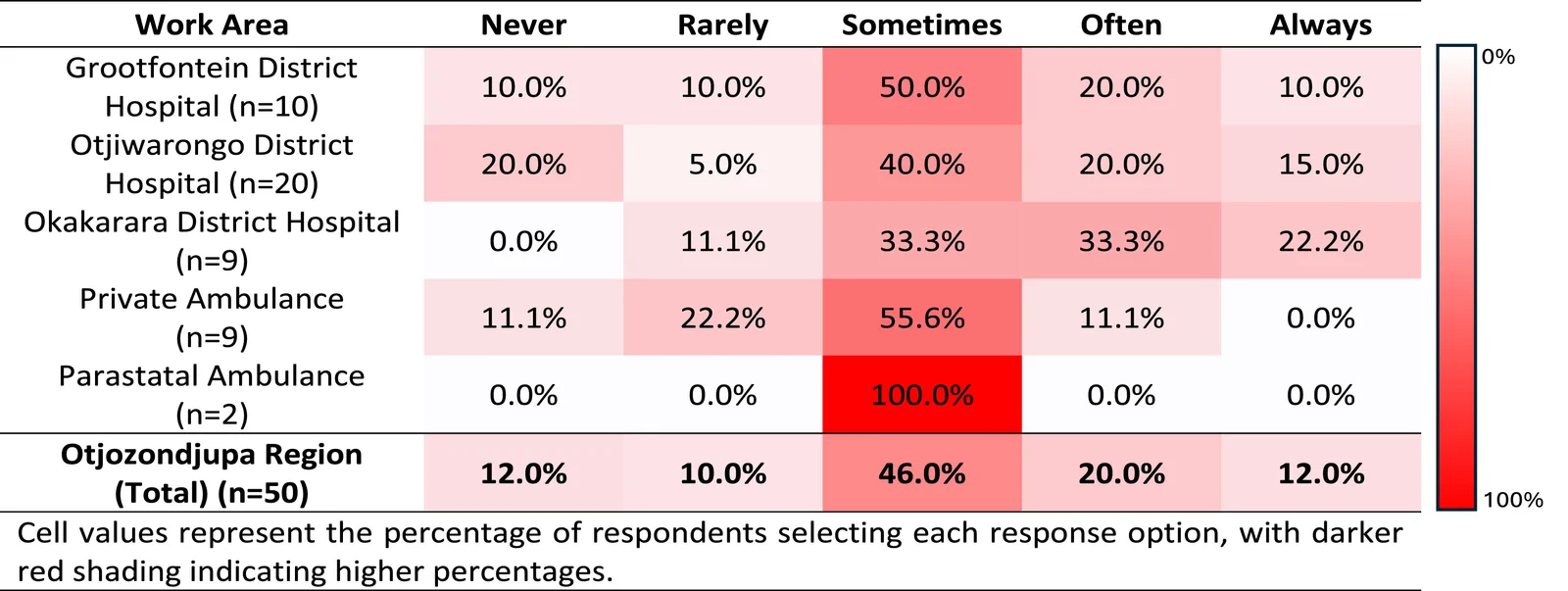
Frequency of referral-transfer pathway delays across medical facilities/ambulance services.

### Clinical factors affecting referral-transfer pathway

#### Diagnostic imaging

Diagnostic imaging (n = 33, 66.0%) was the most frequently reported clinical contributor to referral delays (sometimes, often, and always), with time to completion of tests prolonged after hours (n = 33, 66.0%). Delays were most reported at ODH (n = 16, 80.0%), followed by GDH (n = 6, 60.0%) (Table 2).

**Table 2:** Respondents’ perceptions of delays related to emergency blood, diagnostic imaging and laboratory diagnostics (n = 50).

Response Category/reported reason	Emergency blood n (%)	Diagnostic imaging n (%)	Laboratory diagnostics n (%)
Perceived frequency of delay (n = 50)			
Never	18 (36.0)	3 (6.0)	13 (26.0)
Rarely	17 (34.0)	13 (26.0)	15 (30.0)
Sometimes	10 (20.0)	29 (58.0)	16 (32.0)
Often	2 (4.0)	4 (8.0)	3 (6.0)
Always	0 (0.0)	0 (0.0)	3 (6.0)
Not Applicable	3 (6.0)	1 (2.0)	0 (0.0)
Time to completion during working hours (n = 50)			
>2 h	N/A	0 (0.0%)	1 (2.0%)
1–2 h	N/A	2 (4.0%)	6 (12.0%)
30–60 min	N/A	9 (18.0%)	27 (54.0%)
<30 min	N/A	39 (78.0%)	16 (32.0%)
Time to completion after hours/weekends (n = 50)			
>2 h	N/A	1 (2.0%)	4 (8.0%)
1–2 h	N/A	13 (26.0%)	15 (30.0%)
30–60 min	N/A	33 (66.0%)	27 (54.0%)
<30 min	N/A	3 (6.0%)	4 (8.0%)
Reported reasons for diagnostic service delays (n = 50)			
Non-functional equipment	N/A	19 (38.0)	1 (2.0)
Technician not immediately available	N/A	21 (42.0)	6 (12.0)
No driver available	N/A	26 (52.0)	9 (18.0)
Long travel distance to collect staff	N/A	8 (16.0)	0 (0.0)
Overtime payment issues	N/A	1 (2.0)	1 (2.0)
Staff shortage	N/A	1 (2.0)	0 (0.0)
Long waiting periods after hours	N/A	0 (0.0)	24 (48.0)
Lack of disposable equipment/stock	N/A	0 (0.0)	1 (2.0)
No available vehicles	N/A	4 (8.0)	0 (0.0)
Not applicable	N/A	0 (0.0)	18 (36.0)
Medical Facility/Service Delays (Refers to respondents who selected “sometimes”, “often” or “always”)			
Grootfontein District Hospital (n = 10)	1 (10.0)	6 (60.0)	2 (20.0)
Otjiwarongo District Hospital (n = 20)	7 (35.0)	16 (80.0)	8 (40.0)
Okakarara District Hospital (n = 9)	2 (22.2)	5 (55.6)	6 (66.7)
Private Ambulance Services (n = 9)	1 (11.1)	4 (44.4)	5 (55.6)
Parastatal ambulance services (n = 2)	1 (50.0)	2 (100.0)	1 (50.0)

*Note:* Percentages are calculated using all respondents as the denominator (N = 50), except in the work-area section, where percentages are calculated using the number of respondents from each work area.

No statistically significant difference was found between a perceived delay in diagnostic imaging (never, rarely, sometimes, often and always) and the medical facility (χ2 = 18.675, df = 16, p = 0.286). Respondents identified driver availability for radiographer transport (n = 26, 52.0%), radiographer availability (n = 21, 42.0%) and non-functional x-ray department (n = 19, 38.0%) as perceived delays. Twenty-eight (56.0%) believed critically injured trauma patients should undergo diagnostic imaging before IFT.

#### Laboratory diagnostics

Laboratory diagnostics were reported less frequently as a delay than diagnostic imaging (n = 22, 44.0% vs n = 33, 66.0%) (Table 2). During standard working hours, 43 (86.0%) reported laboratory tests completed within the first 60 min, vs 31 (62.0%) after hours and on weekends. ODH had the highest proportion of reported delays (n = 8, 16.0%), followed by OKDH (n = 6, 12.0%).

Respondents identified long waiting times for staff after hours (n = 24, 48.0%), driver shortage for sample delivery (n = 9, 18.0%) and no laboratory personnel available (n = 6, 12.0%) as perceived delays in IFT of critically injured trauma patients. A statistically significant difference was found between laboratory diagnostic delay (never, rarely, sometimes, often, and always) and medical facility (χ2 = 29.204, df = 16, p = 0.023).

#### Emergency blood and stabilisation/interventions

Obtaining emergency blood was not identified as a contributor to delays (never or rarely) in IFTs by most participants (n = 35, 70.0%) (Table 2). Stabilisation was ranked first as the most time consuming procedures (n = 24, 48.0%), followed by diagnostics in second (n = 23, 46.0%), emergency blood in third (n = 18, 36%) and patient packaging in fourth (n = 26, 52.0%) Procedures most often cited as causing delays included airway management (n = 20, 40.0%), chest tube insertion (n = 17, 34.0%), and central line access and fracture stabilisation (n = 11, 22.0% each) (Table 2).

### Non-clinical factors affecting referral-transfers pathway

System-related factors perceived by respondents were attributed to ambulance availability (n = 39, 78.0%), no MD at the ED on patient arrival (n = 28, 58.0%), the receiving MD at the referral facility unavailable (n = 28, 56.0%), bed shortages at referral hospital (n = 26, 52.0%), and waiting for drivers and clinical staff to conduct the IFT, reported by 25 (50.0%) and 24 (48.0%) respondents respectively. These factors are particularly relevant in the Otjozondjupa setting, where district hospitals rely on road-based interfacility transfer to referral hospitals in Windhoek over long distances.

### Policy awareness and recommendations

Thirty-one (62.0%) respondents either stated that none existed or were unsure whether a designated trauma team existed in the EC. Similarly, 31 (62.0%) respondents reported being unaware of established trauma care protocols (Table 3). The majority (n = 48, 96.0%) agreed, that trauma care protocols would benefit emergency care.

**Table 3:** Designated trauma teams and internal directives and SOPs for established trauma care protocols.

		GDH (n = 10), n (%)	ODH (n = 20), n (%)	OKDH(n = 9), n (%)	Private Amb (n = 9) n (%)	Parastatal Amb (n = 2) n (%)	Total (n = 50) n (%)
**Designated 24 h trauma team**	Yes	5 (50.0)	5 (25.0)	3 (33.3)	5 (55.0)	0 (0.0)	18 (36.0)
	No	2 (20.0)	11 (55.0)	5 (55.5)	0 (0.0)	1 (50.0)	19 (38.0)
	Don’t know	3 (30.0)	4 (20.0)	1 (11.1)	3 (33.3)	1 (50.0)	12 (24.0)
	N/A	0 (0.0)	0 (0.0)	0 (0.0)	1 (11.1)	0 (0.0)	1 (2.0)
	Total	10 (100.0)	20 (100.0)	9 (100.0)	9 (100.0)	2 (100.0)	50 (100.0)
**Knowledge of Internal directive or SOP of trauma care protocols**	Yes	3 (30.0)	6 (30.0)	3 (33.3)	2 (22.2)	1 (50.0)	15 (30.0)
	No	2 (20.0)	9 (45.0)	4 (44.4)	0 (0.0)	1 (50.0)	16 (32.0)
	Don’t know	5 (50.0)	4 (20.0)	2 (22.2)	4 (44.4)	0 (0.0)	15 (30.0)
	N/A	0 (0.0)	1 (5.0)	0 (0.0)	3 (33.3)	0 (0.0)	4 (8.0)
	Total	10 (100.0)	20 (100.0)	9 (100.0)	9 (100.0)	2 (100.0)	50 (100.0)

Ambulance (Amb), Grootfontein District Hospital (GDH), Otjiwarongo District Hospital (ODH), Okakarara District Hospital (OKDH), Standard Operating Procedure (SOP).

Awareness of the national referral policy was limited, with 37 (74.0%) respondents being unaware. Among those aware, MDs (n = 7, 53.0%) had the highest representation, followed by ENs (n = 3, 25.0%) and RNs (n = 2, 22.0%), utilising the policy actively in clinical decision-making. When asked whether their facility has an existing dispatch policy, 19 (38.0%) reported none, and 18 (36.0%) were unsure.

### Suggested improvements

Suggested improvement clustered into targeted training, improved resource allocation, and enhanced IT systems. Training should address communication, clinical development, and standardised protocols for referral, dispatch, and management. Resources should focus on well-equipped, maintained ambulances and additional staff, including drivers, EMS practitioners, nurses, and specialised regional skills. IT systems should support faster, more accurate diagnosis.

## Discussion

Delayed referral, transfers and access to definitive injury care have been reported in several LMIC settings [10,11,16]. However, the literature does not specifically address critically injured trauma patients. To our knowledge, this study is the first in Namibia to explore healthcare workers' perceptions of clinical and non-clinical factors that delay the referral-transfer pathway of critically injured trauma patients and to elicit potential mitigation strategies. Seventy-eight percent of the participants reported encountering referral and transfer delays at least some of the time, underscoring a pervasive challenge in trauma care pathways in the Otjozondjupa Region.

### Clinical factors

Our findings are consistent with prior studies [15,17,18] demonstrating that diagnostic imaging can delay transfers or ICU admissions, particularly after hours. This time variation is concerning, as traumatic incidents commonly occur outside working hours and on weekends [[19], [20], [21], [22]].

Previous studies [[15], [16], [17]] quantified imaging-related delays, they suggest such delays are a widespread challenge across medical facilities. Our survey adds perceived practical contributors, including radiographer availability, driver availability and non-functional X-ray departments. Understanding these causes may inform targeted new strategies and protocols to mitigate delays. Furthermore, the observed consensus among medical facilities regarding the causes of delays may reflect a broader system issue. Referral-transfer delays may be compounded by perceptions that critically injured patients require diagnostic imaging before transfer [17]. Imaging should be considered in relation to stabilisation needs and the risk of delaying definitive care [17].

Facility processes, such as patient handovers, assessments, and admissions, may further delay definitive management. Although necessary, these processes compound existing delays before surgery and definitive management. Up to one-third of delays can be attributed to medical procedures [15,17], highlighting the need to balance lifesaving interventions and procedures for stabilisation with the need to minimise referral and transfer time [15].

### Non-clinical factors

Non-Clinical factors were mostly system-related issues. Paperwork was not perceived as a primary barrier, unlike some previous studies [23]. However, consistent with other findings [17], delays can reflect the receiving team's readiness. For example, one-third [17] of air-ambulance transfer delays were due to disposition holdups or unassembled teams, while ICU admissions were delayed by bed unavailability [18], reinforcing our finding that shortages of referral-hospital beds are a recurrent obstacle.

Ambulance shortages contributed to delays, aligning with national reports of limited ambulances for patient transport [24]. In this setting, ambulances provide both primary response and non-urgent IFT, leaving critically injured patients frequently without available transport. Human resource gaps also emerged, with delays occurring while waiting for clinical staff or drivers to perform the IFT.

Respondents frequently reported the absence of MDs upon EC arrival and prolonged transfer readiness times. According to most respondents, the district hospitals studied lacked a structured 24-hour trauma team, SOPs, or formalised trauma protocols. However, participants agreed that implementing such protocols would enhance EC efficiency. Evidence shows that well-defined protocols improve clinical decision-making and outcomes, while organised trauma systems can reduce mortality by 15–20% [25,26]. The WHO’s Trauma Care Checklist has proven to improve care quality [27] and provides a ready template for such local protocols. Limited awareness of the 2015 Namibian National Referral Policy suggests gaps in dissemination and training that might affect transfer coordination.

Finally, the absence of quality management programs, including dispatch protocols, was evident across facilities. The absence of formal dispatch and transfer coordination protocols may contribute to delays in the referral and IFT process, as the WHO emergency care guidance identifies transport, referral, communication and system coordination as essential emergency care functions [28]. Timeliness is recognised as a key domain of healthcare quality, underscoring the importance of timely referral, IFT, and access to definitive care [29]. Developing clear dispatch protocols may help minimise transfer delays for critically injured patients.

### Mitigating activities

Standardised management protocols, coupled with short, targeted training, can improve team performance and patient outcomes. Human-resource gaps were a recurrent theme; Namibia’s physician ratio of 6.01 per 10,000 population falls well below the WHO target of 10 per 10,000 population [30], highlighting the need for health workers. Strengthening the workforce promotes on-site coverage and rapid IFT deployment.

Well-equipped and well-maintained ambulances are essential for addressing logistical challenges. Evidence from South Africa supports establishing dedicated, well-resourced IFT services [31]. Routine vehicle checklists enhance readiness by detecting stock depletion and vehicle and equipment failures, while sealed tray systems, successfully trialled in United Kingdom hospitals [32], can be implemented to prevent tampering or reliance on stock checks.

Systems-enhanced diagnostic and communication technologies were viewed as high-impact enablers. Artificial intelligence platforms can accelerate radiographic and laboratory interpretation, matching or exceeding human accuracy in some settings [33], thereby shortening stabilisation intervals. Building local capacity and in-region clinical capabilities would reduce reliance on tertiary transfers altogether. Upskilling district hospital staff and establishing onsite specialist teams could enable definitive care closer to injures, aligning with the MoHSS (2015) [8] directive to equip peripheral facilities for timely, in-situ trauma management.

Collectively, these mitigation strategies offer a multifaceted roadmap, focused on staff empowerment, resource optimisation, and digital innovation, to streamline IFT processes and improve outcomes for critically injured patients.

### Study limitations

This study covered one Namibian region and included respondents from district hospitals and ambulance services involved in referral and IFT from Otjozondjupa to state referral hospitals in Windhoek and was limited to trauma patients. Therefore, the findings may not be generalizable to other Namibian regions, private hospital systems, or settings with different pathways, staffing models or ambulance availability. Some services were represented by a small number of respondents, influencing the relative weighting of perceived barriers reported across facilities. The study relied on respondents' opinions and perceptions; therefore, no quantitative, factual data were obtained. Furthermore, inter-rater reliability may vary due to participants' levels of training, work experience, and background. No specific criteria defined a delay, which contributed to the differing perceptions.

## Conclusion

Healthcare workers in Otjozondjupa perceived IFT delays of critically injured trauma patients as common. Perceived clinical contributors included diagnostic imaging and lengthy medical procedures, underscoring the need to focus on life-saving interventions when necessary. Perceived non-clinical contributors included system-related issues: limited ambulance availability, staffing constraints, receiving facility bed/MD availability and a limited awareness of referral policies and trauma care protocols. These findings highlight areas within the regional trauma-transfer pathway that may warrant local review. Future research should prospectively measure transfer times and objectively assess contributors to delays before implementing policy- or system-level interventions.

## Dissemination of results

The results of this study have not yet been formally disseminated to the relevant stakeholders. The research was conducted and submitted to the Namibia University of Science and Technology (NUST) in partial fulfilment of the requirements for the Bachelor of Emergency Medical Care (Honours) degree.

## Declaration of generative AI and AI-assisted technologies in the manuscript preparation process

During the preparation of this work the author(s) used ChatGPT to proofread, verify and format references and to assist with word reduction. After using this tool/service, the author(s) reviewed and edited the content as needed and take(s) full responsibility for the content of the published article.

## CRediT authorship contribution statement

**Johan Du Plessis:** Conceptualization, Data curation, Formal analysis, Investigation, Methodology, Resources, Software, Visualization, Writing – original draft, Writing – review & editing. **Paulus Ambunda:** Conceptualization, Project administration, Supervision, Writing – review & editing. **Andrit Lourens:** Formal analysis, Investigation, Methodology, Project administration, Software, Supervision, Validation, Writing – review & editing.

## Declaration of competing interest

Andrit Lourens was an associate editor of the African Journal of Emergency Medicine at the time of the peer review; however, was not involved in the editorial process or decision making around this manuscript. The authors have no further conflicts of interest to declare.
