# Supplementary material for: Perceived diagnostic and system-related barriers to timely referral and interfacility transfer of trauma patients in Namibia: a cross-sectional survey
Source: Afr J Emerg Med. 2026 Oct 31;16(4):101026. doi: 10.1016/j.afjem.2026.101026 (PMC13743949; doi:10.1016/j.afjem.2026.101026)
Supplement: Supplementary file 1 [file mmc1.docx]

**Supplemental Material**

**Supplemental Material 1:** Questionnaire

**Factors influencing IFTs of critically injured trauma patients from Otjozondjupa region.**

I am Johan Du Plessis, and I am an enrolled student at the Namibia University of Science and Technology (NUST) studying towards a Bachelor of Emergency Medical Care Honours degree. I am an Advanced Life Support Paramedic employed in Otjiwarongo. We would like to invite you to take part in a research project which involves the completion of an online questionnaire. Your participation is entirely voluntary, and you are free to decline to participate or to stop completing the questionnaire at any time, even if you have agreed to take part initially. However, once you have submitted your completed questionnaire online your data will be anonymised, and you will no longer be able to withdraw as there will be no way of linking you to your responses.
This study aims to determine the clinical and non-clinical factors leading to the delay of interfacility transfers (IFTs) of critically injured trauma patients from the Otjozondjupa district hospitals to Windhoek referral hospitals in Namibia. Participants will be selected from the Grootfontein, Okakarara and Otjiwarongo district hospitals, as well as private, parastatal and state emergency medical services. You are eligible to participate if you are a healthcare worker working in the Emergency Department (ED)/Casualty department for more than 6 months, of the district hospitals in the Otjozondjupa region, including medical officers, registered and enrolled nurses and emergency care practitioners – intermediate life support and higher from private and state services.

Your knowledge and experience are highly valued. Your input can have a significant impact on future developments towards timely IFT and better patient outcomes.

If you agree to participate you will be requested to answer the following questions:

Demographics, clinical factors causing delays in IFTs of critically injured trauma patients, the non-clinical factors causing delays in IFTs of critically injured trauma patients, and measure that may mitigate delays of IFTs of critically injured trauma patients.

The questionnaire would take approximately 15 – 20 minutes to complete and is accessible via any smartphone operating system and will be open from 15 September 2023 - 06 October 2023.
There are no risks associated with the completion of the questionnaire. All data is anonymous, and access is limited to the researcher, supervisor and statistician only with data being stored securely. The research findings may have various benefits such as the identification and mitigation of factors that cause delays in IFTs of critically injured patients, the revision of policies and procedures, and the optimisation of transfer procedures.  

This study received ethical approval from the Faculty Research Ethics Committee of the Namibia University of Science and Technology (FHNRAS:42/2023) as well as permission to conduct research from the Research Committee of the Ministry of Health, and Social Services (Ref:22/3/1/2).

If you have any questions, please contact me at +264 81 624 8989 and [johandp35@gmail.com](mailto:johandp35@gmail.com).

Required

1. Click **START SURVEY**, if you wish to participate in the Survey.

By clicking **START SURVEY** you are confirming that you are: ≥18 years of age; have read and understood the information; you agree to participate and understand that your participation is voluntary. 

Click **DECLINE TO PARTICIPATE**, if you do not wish to participate in the survey (please follow all the prompts).

- Start survey.
- Decline to participate.

**Permission to have all anonymous data shared with journals.**

When this study has been completed, the researcher(s) would like to publish the results of the study in journals. Most journals require us to share your anonymous data with them before they publish the results. Therefore, we would like to obtain your permission to have your anonymous data shared with journals.

2. Tick the option you choose for anonymous data sharing with journals

- I agree to have my anonymous data shared with journals during the publication of the results of this study.
- No, I do not agree to have my anonymous data shared with journals during the publication of the results of this study.

**Demographic Information**

This section records the demographic details of all participants to determine the even distribution of the questionnaire and active participation.

3. Indicate your sex

- Male
- Female

4. Indicate your age group

- 18 - 25 years
- 26 - 35 years
- 36 - 50 years
- >50 years

5. Indicate your profession

- Medical Officer
- Specialist Doctor
- ALS Paramedic
- Registered Nurse
- Enrolled Nurse
- Emergency Care Technician (ECT)
- Intermediate Life Support (ILS)

6. Which department or setting do you work in?

- Casualty / Emergency Department
- Pre-Hospital

7. Time in current position and department/setting

- 6 -12 Months
- 12 - 24 Months
- >24 Months

8. Indicate the medical facility or ambulance service you are currently working for

- Grootfontein District Hospital
- Otjiwarongo District Hospital
- Okakarara District Hospital
- Private Ambulance Services
- State Ambulance Services
- Parastatal Ambulance Services

**Clinical Factors**

This section will focus on clinical factors that have the potential to cause a delay in the IFT of critically injured trauma patients. 

Please answer the questions honestly to the best of your knowledge and experience. If you do not have knowledge of the question, please answer with N/A. Please use the below definitions as a guide to complete the questionnaire. 

**Never** - Have never occurred.
**Rarely** - About 25% of the time
**Sometimes** - At least 50% of the time
**Often** - About 75% or more of the time
**Always** - 100% of the time.

9. Diagnostic imaging (X-Ray, Sonar, CT scan etc) can cause delays in IFT's

- Never
- Rarely
- Sometimes
- Often
- Always
- N/A

10. In your experience, on average, how long does it take from requesting an X-ray to completion of X-rays in critically injured trauma patients during **standard working hours** (Monday to Friday from 08h00 – 17h00)?

- <30 minutes
- 30 -60 minutes
- 1 - 2 hours
- >2 hours

11. In your experience, on average, how long does it take from requesting an X-ray to completion of X-rays in critically injured trauma patients, **after-hours** (Monday to Friday from 17h00 – 08h00, weekends and public holidays)?

- < 30 minutes
- 30 - 60 minutes
- 1 - 2 Hours
- >2 Hours

12. If you have answered rarely, sometimes, often, or always in question 9, what are the causes of delays in medical imaging (you can select more than one answer)

- No Radiographer
- X-Ray department out of order
- No driver available to pick up the radiographer.
- Other: Please specify

13. In your opinion, can a critically injured patient be referred without diagnostic imaging for emergent intervention in referral hospitals?

- Never
- Rarely
- Sometimes
- Often
- Always
- N/A

14. Laboratory Diagnostics can cause delays in the IFT of critically injured trauma patients.

- Never
- Rarely
- Sometimes
- Often
- Always
- N/A

15. In your experience, on average, how long does laboratory diagnostics take from requisition to completion of laboratory results in critically injured trauma patients during **standard working hours** (Monday to Friday from 08h00 – 17h00)?

- <30 minutes
- 30 - 60 minutes
- 1 - 2 Hours
- >2 Hours

16. In your experience, on average, how long does laboratory diagnostics take from requisition to completion of laboratory results in critically injured trauma patients **after-hours** (Monday to Friday from 17h00 – 08h00, weekends and public holidays)?

- < 30 minutes
- 30 - 60 minutes
- 1 - 2 hours
- >2 hours

17. If you answered rarely, sometimes, often, or always in question 14, what are the causes of delay in laboratory testing?

- No driver to deliver blood samples.
- Laboratory personnel not available
- Long waiting periods for laboratory personnel after hours
- N/A

18. Does your facility have access to 24-hour operated laboratories?

- Never
- Sometimes
- Always
- Unsure

19. Do you experience delays in obtaining emergency blood for transfusions?

- Never
- Rarely
- Sometimes
- Often
- Always
- N/A

20. In the order from the longest to the shortest time to perform medical procedures, please rank each procedure performed to stabilize a critically injured patient from 1-8:

21. Related to question 20, please add additional causes if not listed here (ranked as above).

22. In order from the most time consuming to the least, please rank the procedures in order from 1 - 4.

23. Related to question 22, please add additional causes if not listed here (ranked as above).

24. In your opinion, is there any other clinical factors that might contribute to delays of IFTs of critically injured trauma patients, that have not been discussed above? Please elaborate.

**Non-Clinical Factors**

This section will focus on the Non-Clinical factors that have the potential to cause a delay in the IFT of critically injured trauma patients. 

Please answer the questions honestly to the best of your knowledge and experience. If you do not have knowledge of the question, please answer with N/A. Please use the below definitions as a guide to complete the questionnaire. 

**Never** - Have never occurred.
**Rarely** - About 25% of the time
**Sometimes** - At least 50% of the time
**Often** - About 75% or more of the time
**Always** - 100% of the time.

25. Patient documentation and referral documents are causing delays in the IFT of critically injured trauma patients.

- Never
- Rarely
- Sometimes
- Often
- Always
- N/A

26. Inability to reach the receiving Medical Officer is a regular occurrence and causes delays in IFTs of critically injured trauma patients.

- Never
- Rarely
- Sometimes
- Often
- Always
- N/A

27. Waiting for clinical staff to conduct the IFT is a cause of delay in IFTs of critically injured trauma patients.

- Never
- Rarely
- Sometimes
- Often
- Always
- N/A

28. Waiting for a designated driver to conduct the IFT of critically injured trauma patients cause delays.

- Never
- Rarely
- Sometimes
- Often
- Always
- N/A

29. Availability of ambulances is a challenge where I work and is therefore a cause of delays in IFT of critically injured trauma patients.

- Never
- Rarely
- Sometimes
- Often
- Always
- N/A

30. Refuelling of ambulances can be attributed to delays in IFTs.

- Never
- Rarely
- Often
- Sometimes
- Always
- N/A

31. Waiting for food packs for patients and personnel can be attributed to delays in IFTs

- Never
- Rarely
- Sometimes
- Often
- Always
- N/A

32. No beds available at the receiving facility is a factor causing delays in IFTs of critically injured trauma patients.

- Never
- Rarely
- Sometimes
- Often
- Always
- N/A

33. There is no Medical Officer in the Casualty at the time of patient arrival and therefore delays treatment and referrals of critically injured trauma patients.

- Never
- Rarely
- Sometimes
- Often
- Always
- N/A

34. Is there a designated team of medical personnel that have been trained for the management of critically injured trauma patients, on standby 24hours?

- Yes
- No
- I don't know.
- N/A

35. In the absence of a trauma unit/centre at your facility, is there an internal directive or SOP for establishing a trauma care protocol at your facility?

- Yes
- No
- I don't know.
- N/A

36. In your opinion, do you think that the establishment of trauma care protocol within your facility would benefit patient management, and overall improvement and optimization of the emergency department?

- Strongly Agree
- Agree
- Neutral
- Disagree
- Strongly Disagree

37. Are you aware of the National Referral Policy of Namibia that was developed to facilitate IFTs of patients?

- Yes
- No

38. If yes, do you refer to the National Referral Policy for emergency patient referrals?

- Never
- Sometimes
- Always
- N/A

39. When reporting or requesting an IFT do you indicate the urgency of the transfer?

- Never
- Rarely
- Sometimes
- Often
- Always
- N/A

40. Does your facility/company have a policy in place indicating the maximum dispatch time from notification of the patient?

- Yes
- No
- Not Sure

**Mitigating Activities**

This section will focus on the mitigating factors that can be implemented to decrease the incidences of delays in IHTs of critically injured trauma patients. 

Please use the below definitions as a guide to complete the questionnaire. 

**Never** - Have never occurred.
**Rarely** - About 25% of the time
**Sometimes** - At least 50% of the time
**Often** - About 75% or more of the time
**Always** - 100% of the time

41. In your opinion, have you encountered one or the other causes of delays as discussed above, in IFTs of critically injured trauma patients from the Otjozondjupa district hospitals to the Windhoek referral hospitals?

- Never
- Rarely
- Sometimes
- Often
- Always

42. In your opinion, what measures can be implemented to decrease the delays of clinical factors in IFTs? Please elaborate.

43. In your opinion, what measures can be implemented to decrease the delays of non-clinical factors in IFTs? Please elaborate.
